# 

**Published:** 1896-03-14

**Authors:** 


### CONTENTS.

**Municipal Dwellings for Working Men** - 389

**The Vaccination Commission** - 390

**Clinical Recollections.** By Sir B. W. RICHARDSON, M.D. - 391

**Is Crime Decreasing?** - 392

**Medical Progress and Hospital Clinics**—

The Leucocyte in Disease. By H. S. WANSBROUGH JONES - 395

PROGRESS IN MEDICINE.—Diseases of the Nervous System - 396

PROGRESS IN SURGERY.—Surgery of the Œsophagus and Stomach - 398

THERAPEUTIC NOTES - 399

NEW APPLIANCES AND THINGS MEDICAL - 400

**Annotations.**—The Secret of the Savoury Sausage—The New Photography in Court—Typhoid and Tenancy: Another Legal Decision - 393

**Notes and News** - 394

**The Institutional Workshop**—

HOSPITAL EXPENDITURE: THE COMMISSARIAT. XI.—The Cost of the Patient - 401

HOSPITAL MEETINGS - 402

CONSTRUCTION NOTES - 404

**Scraps and Gleanings** - 404

**The Book World of Medicine and Science** - 405

### EXTRA SUPPLEMENT.—THE NURSING MIRROR.

**News from the Nursing World.**—H.R.H. Princess Maud's Wedding Present—A Generous Gift—Royal Portsmouth Hospital—Good Service Recognised—A Wise Poor Law Inspector—Help from the Chapels—Queen's Nurse at Stamford—St. Michael's Convalescent Home, Westgate—Food, Dressings, and Dignity—Medical Missions in India—Short Items - ccvii

**Midwifery Papers.** I.—The Female Pelvis - ccix

**Royal British Nurses' Association** - ccix

**Royal National Pension Fund for Nurses** - ccx

**East London Nursing Society** - ccxi

**Private Nursing.** III.—Private Nurses' Attitude towards the Doctor - ccxi

**Pleasant Quarters for Nurses** - ccxii

**Training School Registries.** I.—School and Protective - ccxiii

**The Trained Nurse.—An American View** - ccxiii

**Doctors and Nurses at Cork** - ccxiv

**Bournville—A Cocoa Colony** - ccxiv

**Everybody's Opinion** - ccxv

**Notes and Queries** - ccxv

**For Reading to the Sick** - ccxvi

**The Book World for Women and Nurses** - ccxvi

